# PD‐1/PD‐L1 inhibitors‐based treatment for advanced renal cell carcinoma: Mechanisms affecting efficacy and combination therapies

**DOI:** 10.1002/cam4.4190

**Published:** 2021-08-12

**Authors:** Lei Ding, Hui yu Dong, Tian ren Zhou, Yu hao Wang, Tao Yan, Jun chen Li, Zhong yuan Wang, Jie Li, Chao Liang

**Affiliations:** ^1^ Department of Urology The First Affiliated Hospital of Nanjing Medical University Nanjing China

**Keywords:** advanced renal cell carcinoma, cellular immunity, combination therapy, fundamental mechanisms, PD‐1, PD‐L1 inhibitor

## Abstract

With the widespread use of PD‐1/PD‐L1 monoclonal antibodies (mAbs) in the treatment of multiple malignant tumors, they were also gradually applied to advanced renal cell carcinoma (aRCC). Nowadays, multiple PD‐1/PD‐L1 mAbs, such as nivolumab, avelumab, and pembrolizumab, have achieved considerable efficacy in clinical trials. However, due to the primary, adaptive, and acquired resistance to these mAbs, the efficacy of this immunotherapy is not satisfactory. Theories also vary as to why the difference in efficacy occurs. The alterations of PD‐L1 expression and the interference of cellular immunity may affect the efficacy. These mechanisms demand to be revealed to achieve a sustained and complete objective response in patients with aRCC. Tyrosine kinase inhibitors have been proven to have synergistic mechanisms with PD‐1/PD‐L1 mAb in the treatment of aRCC, and CTLA‐4 mAb has been shown to have a non‐redundant effect with PD‐1/PD‐L1 mAb to enhance efficacy. Although combinations with targeted agents or other checkpoint mAbs have yielded enhanced clinical outcomes in multiple clinical trials nowadays, the potential of PD‐1/PD‐L1 mAbs still has a large development space. More potential mechanisms that affect the efficacy demand to be developed and transformed into the clinical treatment of aRCC to search for possible combination regimens. We elucidate these mechanisms in RCC and present existing combination therapies applied in clinical trials. This may help physicians’ select treatment options for patients with refractory kidney cancer.

## Graphical abstract

PD‐1/PD‐L1 inhibitors have been gradually applied in the treatment of advanced renal cell carcinoma, but the efficacy is limited. Alternations in PD‐L1 expression and cellular immunity can directly or indirectly affect the efficacy of PD‐L1 inhibitors. Antiangiogenic drugs and CTLA‐4 inhibitors have been shown to significantly improve the efficacy of PD‐1/PD‐L1 inhibitors.

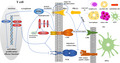

## 1 INTRODUCTION

Kidney cancer is the sixth and eighth most common cancer in men and women in 2020, accounting for approximately 5% and 3%, respectively, according to estimates by the American Cancer Society.^1^ In accordance with tumor histology and chromosomal alterations, kidney cancer is mainly classified into clear cell renal cell carcinoma (ccRCC), chromophobe RCC, papillary RCC, translocation RCC, and other rare subtypes of the renal unit or collection system. CcRCC is the main type, accounting for approximately 70%.^2^ The 5‐year survival rate for localized kidney and renal pelvic cancer was 92.6%, and that for regional cancer was only 66.7%. This rate even fell to 11.7% in patients with extensive metastatic cancer.^3^ Therefore, the development of effective and safe agents for aRCC is urgent.

Through these years, therapies for patients with aRCC who are ineligible for partial or radical nephrectomy have undergone a series of revolutions, from the initial radiotherapy, chemotherapy, to nonspecific immunotherapy, such as interleukin‐2 and interferon, and then to targeted therapies, such as vascular endothelial growth factor (VEGF) inhibitors and mTOR inhibitors. Despite the impressive progress, the objective response rates (ORRs) and safety of these agents remain unsatisfactory.^4^ Due to the high immunogenicity and strong T cell infiltration of RCC,^5^, ^6^ programmed cell death‐1/ programmed cell death‐ligand 1 (PD‐1/PD‐L1) monoclonal antibodies (mAbs), which can strengthen cellular immunity, have been gradually applied in the treatment of aRCC as monotherapy recently and achieved considerable efficacy and acceptable safety, especially in ccRCC.^7^ However, its ORRs as first‐line therapy were only around 16%–37%, which still could not bring clinical benefits to most patients.^8^, ^9^, ^10^, ^11^ In this instance, the combination of multiple checkpoint inhibitors or PD‐1/PD‐L1 mAbs combined with antiangiogenic agents (AAs) has emerged, whose efficacy was superior to the above targeted agents, and no statistical difference exists between them in terms of safety. In these clinical trials, the ORRs of the combination regimens could increase to approximately 59%.^12^, ^13^, ^14^, ^15^, ^16^ However, a proportion of patients still has no response, and safety issues could not be ignored.

Here, the underlying mechanisms affecting the efficacy of PD‐1/PD‐L1 mAbs in aRCC; the therapies that could be combined with PD‐1/PD‐L1 mAbs, including chemotherapy, radiotherapy, and vaccine; and the existing related clinical trials were reviewed to develop novel combined therapeutic targets and potential predictive markers for efficacy. This review may be helpful for the development of new combination therapies for aRCC.

## 2 PRECLINICAL STUDIES OF PD‐1/PD‐L1 MABS AND ITS LANDSCAPE IN THE TREATMENT OF ARCC

PD‐1 is a 288 amino acid (aa) type I transmembrane glycoprotein encoded by *PDCD1* on human chromosome 2, whose cytoplasmic domain includes an immunoreceptor tyrosine‐based inhibitory motif (ITIM) and an immunoreceptor tyrosine‐based switch motif. As for PD‐L1, the ligand for PD‐1 is a 290 aa type I transmembrane glycoprotein encoded by *CD274* on human chromosome 9.^17^, ^18^ After the activation of T cells, PD‐1 expression is upregulated within 12–36 h to interact with PD‐L1 to inhibit the function of T cells via various mechanisms and prevent indiscriminate killing of excessive activated T cells to normal cells.^19^ However, PD‐L1 is not only expressed on lymphocytes, myeloid, and endothelial cells but also on tumor cells, tumor infiltrating lymphocytes (TILs), macrophages, and other immune cells in the tumor microenvironment (TME).^20^ Therefore, PD‐1/PD‐L1 axis has also been revealed to participate in mediating antitumor immunity, and the overexpression of PD‐1/PD‐L1 signaling pathway could influence the cytolytic activity of T cells and thus promote occurrence and invasiveness of tumors.^21^, ^22^

**FIGURE 1:**
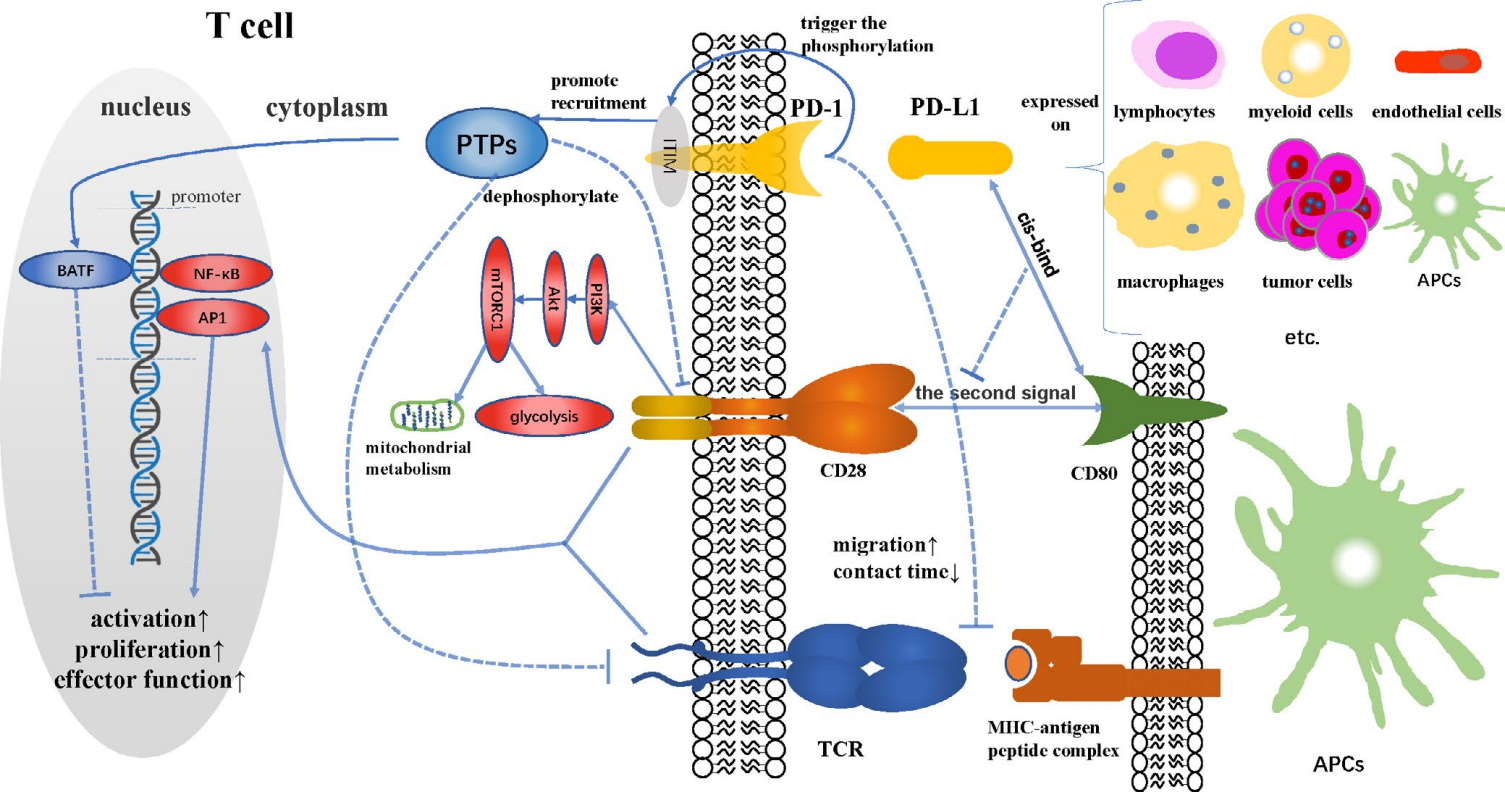
PD‐L1 expressed on tumor cells, antigen‐presenting cells and other cells can interact with PD‐1 on T cells to inhibit the proliferation, activation, metabolic activity, and effector function of T cells by affecting costimulatory molecules, transcription factors and antigen presentation, and finally induce tumor immune resistance

The interaction between PD‐1 and PD‐L1 could trigger the phosphorylation of tyrosine residues in ITIM from PD‐1 and promote the recruitment of protein tyrosine phosphatases (PTPs), such as SHP2 and PP2A. These PTPs dephosphorylate TCR^23^; costimulatory molecules, such as CD28,^24^ on the surface of T cells; and stimulant molecules downstream of related signaling pathways, resulting in decreased activation of transcription factors, such as activating protein 1 and NF‐κB.^25^, ^26^, ^27^ Blocking the costimulatory effect of CD28 could downregulate the downstream PI3K/Akt/mTORC1 signaling pathway, which not only inhibits glycolysis, but also induces the formation of intracellular mitochondrial crest and impairs oxidative phosphorylation, thereby inhibiting the metabolic activity of CD8+ T cells.^28^, ^29^ Moreover, the PD‐L1 expressed on antigen‐presenting cells (APCs) could *cis*‐bind to CD80, a costimulatory molecule on the surface of these cells, thus blocking PD‐1 ligation and the stimulation of CD28/CD80 to T cells.^30^, ^31^ The expression of BATF, a transcription factor that inhibits T‐cell activation, could also be induced by PD‐1.^32^ In addition, the engagement of PD‐1 could enhance the migration ability of T cells and reduce the contact time between T cells and the major histocompatibility complex (MHC)–antigen peptide complex on interacting cells.^33^ Ultimately, the above mechanisms block the function of T cells by antagonizing T‐cell activation, proliferation, and effector function (Figure 1).

The mechanisms by which these tumors resist endogenous tumor‐specific T cell killing via the PD‐1/PD‐L1 axis could be divided into two types: intrinsic and adaptive immune resistance, which are compatible and could coexist within the same TME. Intrinsic immune resistance is associated with alterations at the level of genes or certain signaling pathways in tumor cells, which could induce constitutive PD‐L1 expression. For example, in RCC, 9p24.1 amplification could directly stimulate constitutive PD‐L1 expression or indirectly stimulate it through the activation of the JAK2 signaling pathway.^34^ Activation of Akt and STAT3 signaling has also been shown to induce constitutive PD‐L1 expression in multiple tumors and participate in immune resistance.^35^, ^36^ Adaptive resistance is mainly induced by cytokines secreted by tumor cells and other cells in TME, especially IFN‐γ.^35^, ^37^ This mechanism is demonstrated as the manifestation of tumors to the stimulation of the immune microenvironment, and it induces adaptive PD‐L1 expression.

Given the above preclinical mechanism, researchers have revealed various methods, such as PD‐1/PD‐L1 mAbs, vaccination, and adoptive immunotherapy, to reverse tumor immune escape by targeting the PD‐1/PD‐L1 axis or interfering with the PD‐1 signaling pathway.^38^, ^39^ In the present review, ClinicalTrials.gov was searched for clinical trials with PD‐1/PD‐L1 mAb monotherapy as an intervention. As shown in Table 1, multiple PD‐1/PD‐L1 mAbs as monotherapy have shown impressive efficacy in clinical trials related to RCC; however, most patients still do not have any immune response, and their security issues should not be underestimated.^8^, ^9^, ^10^, ^11^, ^40^, ^41^, ^42^, ^43^, ^44^, ^45^ Some patients also experienced drug resistance during treatment, further eroding the overall efficacy. The underlying mechanism of drug resistance should be identified to address the problem of low ORRs.

**TABLE 1:** Clinical trials using PD‐1/PD‐L1 mAbs as intervention

NCT, study	Phase	Setting	Experimental arm (pts,agents)	Control arm (pts,agents)	Primary endpoints	ORR(%)	Serious AEs (exp,%)
NCT00730639 CA209‐003	I	First line; CRPC,RCC,MM, NSCLC.	18 (1.0 mg/kg, arm1) 16 (10.0 mg/kg, arm2) nivolumab	None	Number of participants With SAEs, TRAEs	NA	NA
NCT01354431 CA209‐010	II	Second line; aRCC,mRCC	60 (0.3 mg/kg, arm1) 54 (2.0 mg/kg, arm2) 54 (10.0 mg/kg, arm3) nivolumab	None	PFS	arm1:20.0, 80%CI13.4–28.2 arm2:22.2, 80%CI15.0–31.1 arm3:20.4, 80%CI13.4–29.1	arm1: 45.76% arm2: 61.11% arm3: 40.74%
NCT01358721 CA209‐009	I	First or second line; RCC	Previously‐treated: 22 (0.3 mg/kg, arm1) 22 (2.0 mg/kg, arm2) 23 (10.0 mg/kg, arm3) Treatment‐naïve: 24 (10.0 mg/kg, arm4) nivolumab	None	Percent change from baseline in Activated and memory T Cells	arm1: 9.1% arm2: 18.2% arm3: 21.7% arm4: 4.2%	arm1: 59.09% arm2: 50.00% arm3: 52.17% arm4: 54.17%
NCT01668784 CheckMate 025	III	Second line; aRCC,mRCC	410 (arm1) nivolumab	411 (arm2) everolimus	OS,PFS,TRAEs (%)	arm1: 25.1,95%CI21.0–29.6 arm2: 5.4, 95%CI3.4–8.0	arm1: 47.78% arm2: 43.58%
NCT02212730 KEYNOTE 031	I	First line; RCC	6 (arm1,pre‐resection) pembrolizumab	4 (arm2,post‐resection) pembrolizumab	AEs during the neoadjuvant pembrolizumab regimen (number)	None	arm1: 33.33% arm2: 0.00%
NCT02596035 CheckMate 374	IV	Second line; aRCC,mRCC	142 nivolumab	None	3 or higehr grade IMAEs (%)	None	41.55%
NCT03444766 CA209‐887	IV	Second line; aRCC,mRCC, NSCLC	100 (overall) nivolumab	None	TRAEs (number)	None	30.00%
NCT01772004 JAVELIN Solid Tumor	I	First or second line; RCC	62 (line1,arm1) 20(line2,arm2) avelumab	None	OS,PFS	arm1: 16.1, 95%CI 8.0–27.7 arm2: 10.0, 95%CI 1.2–31.7	arm1: 22.6% arm2: 35.0%
NCT02836795 Junshi‐JS001‐BJZL‐I	I	First or second line; RCC,M,UC	6 (RCC) toripalimab	None	TRAEs(all grade, 3 or higher grade), ORR	33.3% (RCC)	13.89%(overall)
NCT01375842 PCD4989g	I	First or second line; aRCC,mRCC	70 atezolizumab	None	TRAEs(all grade, 3 or higher grade)	15%, 95CI 7–26%	NA
NCT00729664 CA210‐001	I	First or second line; RCC,NSCLC,etc.	17 (RCC) MDX 1105 (PD−1 mAb)	None	TRAEs(all grade, 3 or higher grade), ORR	12%, 95CI 2–36 (RCC)	5%(overall)
NCT02853344 KEYNOTE−427	II	First line; RCC	110 (arm1: ccRCC) 165 (arm2: nccRCC) pembrolizumab	Nnoe	ORR	arm1: 36.4%, 95% CI 27.4–46.1 arm2: 26.7%, 95% CI 20.1–34.1	High‐grade TRAEs: arm1: 30% arm2: 17%

Clinical trials with PD‐1/PD‐L1 mAb monotherapy as an intervention that had results and enrolled more than 5 patients with RCC. The characteristics and partial results of the these clinical trials are presented in the table.

Abbreviations: aRCC, advanced renal cell carcinoma; ccRCC, clear cell renal cell carcinoma; CI, confidence interval; CRPC, causation resistant prostate cancer; DCR, disease control rate; DOR, duration of response; M, melanocytoma; MM, malignant melanocytoma; mRCC, metastatic renal cell carcinoma; NA, not available; NE, not estimate; NSCLC, non‐small cell lung cancer; OS, overall survival; PFS, progression free survival; pts, patients; SAEs, serious adverse events; TRAEs, treatment‐related adverse events; UC, urothelial cancer

## 3 MECHANISMS OF PD‐L1 EXPRESSION ALTERATIONS IN RCC

**FIGURE 2:**
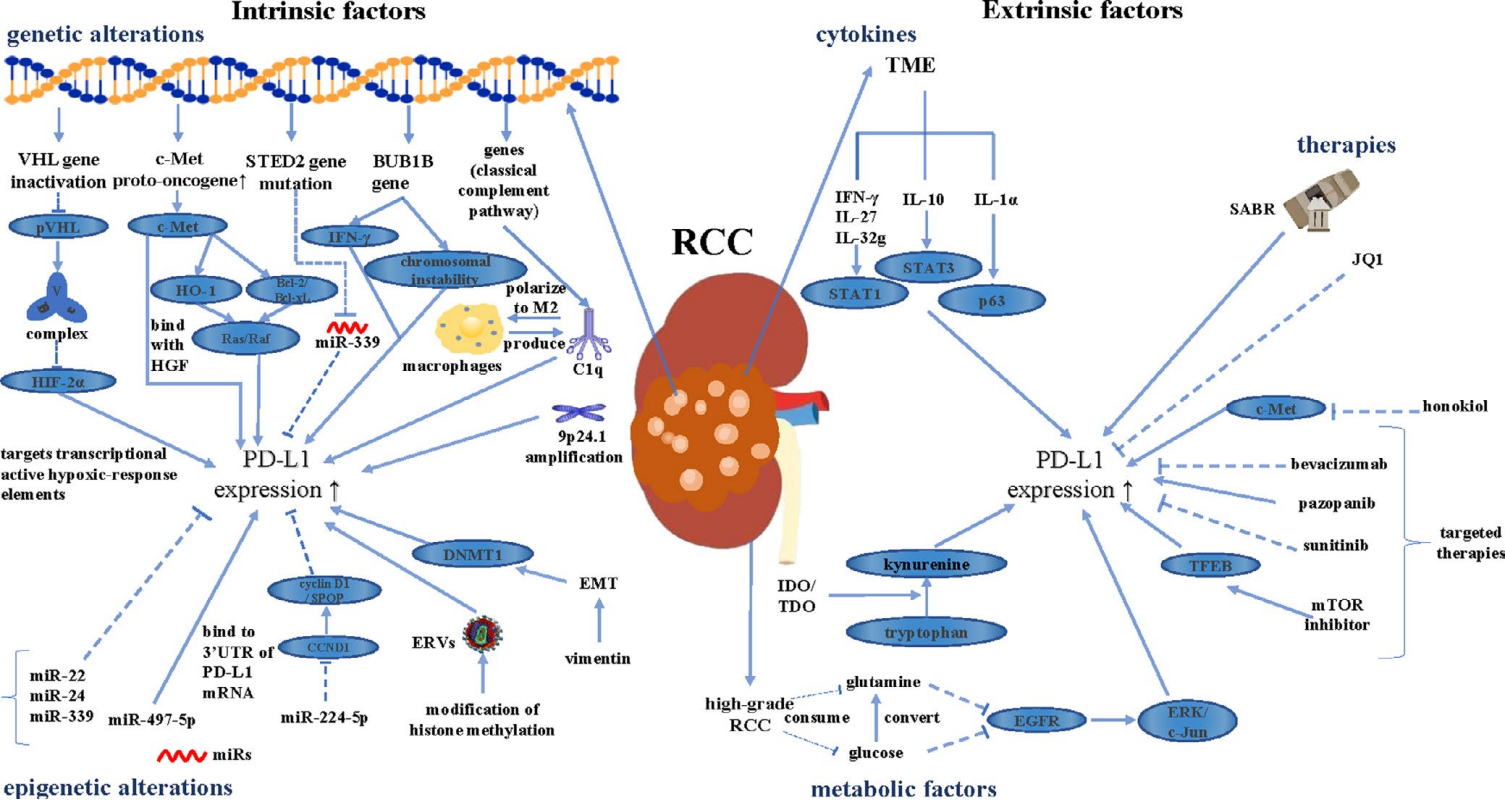
The alterations of tumor cells themselves, tumor microenvironment and tumor external factors can affect the expression of PD‐L1 in TME of RCC. Among them, genetic alterations, microRNA networks and clinical treatment have significant influence on PD‐L1 expression in RCC. In addition, the effect of anti‐angiogenic drugs on the expression of PD‐L1 is not uniform, and these potential mechanisms still need to be further explored

Researchers initially found that the high level of PD‐L1 expression on tumor cells or TILs in TME was often accompanied by increased TMN stage and cell atypia in RCC, which also indicates increased risk of disease progression and worsened prognosis.^46^ In two clinical trials, CheckMate214 and KEYNOTE426, no significant difference was demonstrated in OS between PD‐L1‐negative patients and PD‐L1‐positive patients.^13^, ^47^ In Javelin Renal 101, no statistically significant difference was found in PFS between these two subgroups.^48^ Thus, PD‐1/PD‐L1 mAbs may be occupied by PD‐L1 expressed on normal cells, such as lymphocytes, myeloid cells, and endothelial cells, and this hypothesis is also the possible reason for the occurrence of immune‐related adverse events. Therefore, more targets of PD‐L1‐positive patients could not show enhanced clinical benefits. Although the application of PD‐L1 expression has many limitations, such as temporal and spatial heterogeneity, measurement techniques, and uncertain positive cutoff value,^49^, ^50^, ^51^ the mechanisms of alterations in its expression are still worth exploring. Based on these potential preclinical mechanisms (Figure 2), researchers could develop other agents that could inhibit the PD‐1/PD‐L1 axis to synergistically enhance the efficacy of PD‐1/PD‐L1 mAbs. Some agents with strong antitumor effect could also induce PD‐L1 expression and lead to immunosuppression. The nonoverlapping effect of PD‐1/PD‐L1 inhibitors and these agents could enhance the antitumor effect.

### 3.1 Intrinsic factors of PD‐L1 expression alteration

Genetic and epigenetic alterations are the main intrinsic factors. Here, these abnormalities that have been demonstrated to affect the constitutive PD‐L1 expression in RCC were described.

#### 3.1.1 Genetic alterations

The biallelic von Hippel Lindau (*VHL*) gene inactivation is the most characteristic genetic mutation in ccRCC; it causes the decrease in the level of VHL protein (pVHL).^52^ Together with Elongin B and Elongin C, pVHL could form VBC complex, a ubiquitin ligase that mediates the hydrolysis of hypoxia‐inducible factor‐α (HIF‐α) in the proteasome pathway.^53^ HIF‐2α targets transcriptional active hypoxic‐response elements in the proximal promoter region of PD‐L1; thus, the accumulation of HIF‐2α caused by VHL inactivation could directly cause constitutive PD‐L1 overexpression.^54^, ^55^ In addition, c‐Met encoding receptor tyrosine kinase, which is overexpressed in clear cell and papillary RCC,^56^, ^57^ could induce PD‐L1 expression at the transcriptional level after binding with its ligand hepatocyte growth factor. This signaling pathway could also upregulate heme oxygenase‐1 (HO‐1) expression and Bcl‐2/Bcl‐xL signaling pathways and ultimately activate the Ras/Raf pathway. This induction mechanism could be reversed by c‐Met inhibitor and weakened by the downregulation of the Ras‐PI‐3K signaling pathway or HO‐1.^58^ In patients with aRCC treated with sunitinib, the positive correlation between c‐Met expression and PD‐L1 expression was also confirmed.^59^ *STED2* encoding methyltransferase is also a common mutation gene in ccRCC.^60^ It could mediate the loss of miR‐339, thus upregulating PD‐L1 expression and weakening antitumor immunity.^61^ Another study that included 121 patients with RCC, suggested that RCC cells overexpressed *BUB1B* in approximately 25% of patients, and these cells had poor response to nivolumab. Data analysis showed that *BUB1B* overexpression was positively associated with PD‐L1, IFN‐γ, and CD8+ T cell exhaustion signals and resulted in upregulation of CD44, phosphorylation of p53, and chromosomal instability. The high IFN‐γ expression and DNA damage caused by chromosomal instability may be the reasons for the upregulation of PD‐L1 expression.^62^, ^63^

Genes encoding classical complement pathway protein were confirmed to be highly expressed in ccRCC, and the C1q encoded by these genes was positively correlated with PD‐L1/PD‐L2 expression.^64^ C1q is mainly produced by the M2 subtype of tumor‐associated macrophages in ccRCC, and it induced macrophages to polarize to immunosuppressive phenotype (M2) in vitro.^64^, ^65^ This interaction is possibly related to the upregulation of PD‐L1/PD‐L2. Amplification of JAK2, PD‐L1, and PD‐L2 at 9p24.1 could often be found in the sarcomatoid tissue of chromophobe or ccRCC.^66^, ^67^ It could directly cause PD‐L1/PD‐L2 overexpression and the upregulation of the JAK2/STAT3 pathway,^34^ which stimulates PD‐L1 overexpression in multiple other tumors by binding its downstream molecule IRF1 to the PD‐L1 promoter.^68^, ^69^

#### 3.1.2 Epigenetic alterations

The microRNA (miRNA) network plays an important role in the regulation of PD‐L1 expression in aRCC, although the underlying mechanisms are not fully understood. A study comparing miRNA expression in 23 patients with metastatic ccRCC before and after treatment with nivolumab showed a negative association between miR‐22, miR‐24, and soluble PD‐L1 expression.^70^ Among patients with persistent response to immunotherapy, those with high miR‐339 expression had better PFS,^70^ and this finding is consistent with that of the aforementioned study.^61^ Another study found that in RCC, miR‐497‐5p could directly bind to the 3′ UTR of PD‐L1 mRNA to inhibit PD‐L1 expression at the protein level.^71^ Su Zeng et al. demonstrated that the miR‐224‐5p expression in urinary extracellular vesicles was abnormally elevated in patients with RCC, and this elevation could inhibit the gene *CCND1* encoding cyclin D1 and thereby downregulate the proteolytic hydrolysis of PD‐L1 mediated by the downstream cyclin D1/SPOP signaling pathway.^72^

Genetic alterations induced by the modification of histone methylation could affect PD‐L1 expression by upregulating the expression of immunogenic endogenous retroviruses (πERVs) in RCC, especially ERV3‐2.^73^ Researchers also found that high vimentin expression in RCC was accompanied by high PD‐L1 expression.^74^ Previous studies suggested that high vimentin expression represented more epithelial–mesenchymal transformation(EMT), which could induce the demethylation of PD‐L1 promoter by upregulating DNA methyl‐transferase 1(DNMT1).^75^, ^76^ Vimentin could also interact with deacetylated PD‐L1 to facilitate its nuclear translocation via cytoskeleton, thus promoting the formation of positive feedback of PD‐L1 expression.^77^ These findings may be the reasons why vimentin is positively correlated with PD‐L1 in RCC.

### 3.2 Extrinsic factors of PD‐L1 expression alteration

The cytokines present in TME could affect PD‐L1 expression, and their high expression is mainly due to the stimulation of RCC by the immune microenvironment. Agents, some other therapies, and metabolic imbalance are also associated with the expression of PD‐L1 in the TME of RCC.

#### 3.2.1 Cytokines

IFN‐γ has been demonstrated to induce PD‐L1 expression in multiple tumors.^78^, ^79^ It is consistent with the positive relationship between PD‐L1 and IFN‐γ in RCC.^80^ IFN‐γ could induce PD‐L1 overexpression at the transcriptional level by promoting STAT1 phosphorylation, which is also the mechanism of IL‐27 and IL‐32g inducing PD‐L1 overexpression.^37^ Researchers revealed that IL‐10 and IL‐1α could promote the phosphorylation of STAT3 and tumor suppressor gene *p65*, respectively, to upregulate PD‐L1 expression.^37^ In melanocytes, the agents that inhibit eukaryotic cell initiation factor 4A could downregulate the transcription level of STAT1 and indirectly downregulate PD‐L1 expression to induce tumor regression.^81^ This mechanism may also be applied in the treatment of RCC to enhance the efficacy of anti‐PD‐1/PD‐L1.

#### 3.2.2 Therapies

Traditional targeted therapy plays an important role in the treatment of RCC.^4^ Eric et al. demonstrated that sunitinib and bevacizumab increased the infiltration level of CD8+ T cells in TME and induced their secretion of IFN‐γ in RCC, thus upregulating PD‐L1 expression.^82^ Studies on thymus‐free nude mice with renal carcinoma have also shown that these AAs could upregulate the PD‐L1 protein levels independent of CD8+ T cells, and experiments in vitro suggested that this mechanism is not affected by HIF‐1/2α.^82^ These findings indicated that AAs may directly induce PD‐L1 expression. However, pazopanib, another AA, downregulates the PD‐L1 expression of dendritic cells (DCs) in aRCC.^83^ The influence of AAs on PD‐L1 expression is still not fully understood. MTOR inhibitors, another type of classic targeted agents, have been revealed to be involved in the regulation of PD‐L1 expression.^84^ They could induce the nuclear translocation of transcription factor EB (TFEB), the main target of mTORC1,^85^ and upregulate its expression. TFEB could bind with PD‐L1 promoter, stimulate PD‐L1 expression, and ultimately inhibit the function of tumor infiltrating CD8+ T cells.^86^

Some untargeted agents also regulate PD‐L1 expression in the TME of RCC. For instance, honokiol, a natural product of bisphenol, could inhibit the phosphorylation of downstream molecule Akt and PD‐L1 expression by inhibiting the c‐Met related signaling pathway, thus enhancing the antitumor immune response.^87^ RCC cells treated with the bromine domain inhibitor JQ1 showed reduced proliferation and reduced PD‐L1/PD‐L2 expression, although the exact mechanism is unknown.^88^ In addition, after neoadjuvant stereotactic radiotherapy (Neo‐SABR), the PD‐L1 expression in the tumor thrombus of patients with RCC and the tumor thrombus of the inferior vena cava increased, which may be attributed to the induction of immune‐related inflammatory cytokines.^89^

#### 3.2.3 Metabolic factors

High‐grade RCC tends to represent more metabolic reprogramming, with glucose disorders being the most common. Glucose deficiency could reduce the energy produced by glycolysis pathway and promote downstream ERK/c‐Jun phosphorylation by activating EGFR, thereby enhancing PD‐L1 expression.^90^ In high‐grade RCC, glucose‐converted glutamine is used to alleviate oxidative stress via the glutathione pathway, and the grade of RCC was positively correlated with the expression of glutamine depletion signature.^91^, ^92^ Glutamine deprivation has been demonstrated to induce PD‐L1 expression by stimulating the EGFR/ERK/c‐Jun signaling pathway. Epidermal growth factor could induce glutamine deprivation to promote PD‐L1 expression.^93^ Inhibitors of EGFR/ERK/c‐Jun have been shown to reverse these effects,^93^ which may enhance the efficacy of PD‐1/PD‐L1 mAbs. Besides, tryptophan metabolism is related to the regulation of PD‐L1 expression. Kynurenine is synthesized by the catabolism of tryptophan by indoleamine 2,3‐dioxygenase/tryptophan 2,3‐dioxygenase (IDO/TDO). Researchers revealed that after the treatment of nivolumab in some patients with aRCC, kynurenine was upregulated by enhancing the activity of IDO/TDO; thus, PD‐L1 expression could be induced to counteract the immune stimulation caused by PD‐1 blockade.^94^ Inhibition of this metabolic pathway could improve tumor immune resistance.^95^

## 4 MECHANISMS AFFECTING CELLULAR IMMUNITY IN RCC

The antitumor immune process mainly involves cellular immunity, which could be summarized as follows: antigen presentation, activation, and migration of immune cells and recognition and killing of tumor cells by immune cells. In addition to PD‐L1 expression, interference with cellular immunity could affect the efficacy of PD‐1/PD‐L1 mAbs. A thorough understanding of these mechanisms may be helpful in enhancing the efficacy of PD‐1/PD‐L1 mAbs.

### 4.1 Antigen presentation

Higher tumor mutation burden (TMB) usually means that more tumor neoantigens are producted to present to T cells by MHC proteins.^96^ However, several retrospective analyses showed no significant association between TMB and immunotherapy response in RCC.^97^ The results of a retrospective analysis of 34 patients with aRCC who received PD‐1/PD‐L1 mAbs were consistent with the above conclusions. They also revealed that heterozygosity loss of MHC‐I class genes associated with antigen presentation occurred by as high as 33% in the progression disease group, leading to antigen presentation limitation. In the disease control group, 68.8% of patients were found to have enrichment of DNA repair gene mutations, especially homologous recombinant repair‐related genes, which may be related to the increase in tumor neoantigens and thus enhance antigen presentation.^98^ In ccRCC, the low intratumor heterogeneity, which is the genetic diversity of subclones in a single tumor, could promote the antigen presentation to T cells by enhancing the immune activity of new tumor antigens, the abundance of DCs, and the expression of HLA class I gene, and ultimately improve the response to PD‐1 blockade.^99^ Although these mechanisms are difficult to interfere with, they may serve as biomarkers for predicting the efficacy. Another single‐cell analysis of aRCC tissues treated with atezolizumab showed that in RCC cells and DC subsets with high IL‐8 expression, the expression of genes involved in antigen presentation and processing, including HLA‐C and IFIT3, decreased, and the overexpression of IL‐8 was associated with poor ORR.^100^

Marianna Nuti et al. found that pazopanib could upregulate the HLA‐DR, CD40, and CCR7 expression levels of DCs in aRCC tissues, inhibit their endocytosis, and ultimately promote the activation and enhance the antigen presentation function.^83^ It could also downregulate PD‐L1 expression on DCs and inhibit the secretion of IL‐10 to enhance the stimulation of T cells, thus inducing Th1 type immune response and promoting the increase in circulating CD137+CD4+ T cells,^83^, ^101^ which may be partially attributed to the inhibition of p‐ERK/β‐catenin signals expressed by DCs.^83^, ^102^, ^103^ This phenomenon may be the theoretical basis for improving the response to PD‐1/PD‐L1 mAbs. Previous studies also showed that intestinal microbiota and its products have cross reactivity with tumor neoantigens, which could enhance antigen presentation and stimulate T‐cell activation,^104^, ^105^ while the use of TAB could disrupt intestinal microbiota and was associated with worsened prognosis in patients with aRCC receiving PD‐1/PD‐L1 mAbs.^106^

### 4.2 Direct effects on immune cells actively involved in cellular immunity

During the activation of T cells, alterations in the expression of costimulatory/coinhibitory molecules on the surface could significantly affect the activity of T cells. In studies related to RCC, researchers demonstrated that CD28 co‐stimulation significantly improved the glycolysis and mitochondrial metabolism of CD8+ TLs, thereby improving their mitochondrial and effector functions, possibly by upregulating GLUT3.^107^ Besides, Joel LeMaoul et al. found two mutually exclusive subgroups in the TME of RCC: CD8+ILT2+PD‐1‐TILs and CD8+ILT2‐PD‐1+TILs. CD8+ILT2+PD‐1‐TILs highly express CD51, perforin (PRF1), and Granzyme‐β (GZMB) and secrete more IFN‐γ, which has strong cytotoxicity.^108^ The use of PD‐1/PD‐L1 mAbs may promote the differentiation of CD8+PD‐1+TILs toward highly cytotoxic ILT2+TILs.^41^ However, the interaction of HLA‐G expressed by tumor cells and ILT2 could significantly inhibit the effector function of TILs, and ILT2 blockade could rescue this inhibition,^108^ indicating the possibility of the combination of HLA‐G/ILT2 blockade and PD‐1/PD‐L1 blockade. The largest proportion of CD4+ and CD8+ T cells isolated from RCC was that expressing PD‐1 and LAG‐3, and PD‐1 blockade could significantly upregulate LAG‐3 expression. In‐vitro experiments showed that the dual blockade of LAG‐3 and PD‐1 could more effectively induce T cells to secrete IFN‐γ and improve the function of TILs in TME.^109^ Moreover, signal lymphocyte activation molecule (SLAM)F7 expressed on tumor‐associated macrophages (TAMs) could activate the self‐ligand SLAMF7 on T cells in RCC, which could promote the phosphorylation of STAT1/3 and upregulate the expression of costimulatory molecules, such as PD‐1 and T cell exhaustion‐related transcription factors. Eventually, the transformation of CD8+ T cells to terminal exhaustion phenotype is induced.^109^ SLAMF7 inhibition may synergistically enhance the blocking effect of PD‐1 mAbs, thus promoting cellular immunity.

The cytokines in TME are also involved in cellular immunity. Bempegaldesleukin (NKTR‐214), a CD122 (IL‐2 receptor β chain) type IL‐2 agonist, binds primarily to the heterodimer IL‐2βγ and continues to stimulate the IL‐2βγ receptor pathway to provide a sustained stimulus signal, thereby prioritizing the stimulation of effector T cells.^110^ In combination with nivolumab, it could significantly upregulate the expression of genes related to CD45+ lymphocytes, CD8+ T cells, macrophages, and natural killer (NK) cells and promote the TME infiltration of CD8+ T cells.^111^ Streptavidin‐IL‐2 surface‐modified tumor cell vaccine could enhance the killing effect of specific antitumor T cells in RCC, but it could also induce high PD‐1 expression on these cells and upregulate PD‐L1 expression in TME. PD‐1/PD‐L1 mAbs could reverse this immune escape, promote IFN‐γ and IL‐12 expression, and further enhance the cytotoxicity of specific antitumor T cells.^112^ Moreover, glutamine‐addicted ccRCC depletes extracellular glutamine and then upregulates HIF‐1α expression, thereby inducing tumor‐infiltrating macrophages to secrete IL‐23. It could promote IL‐10 and TGF‐β expression and then decrease the IFN‐γ, GZMB, and PRF1 expression levels on CD8+ T cells to inhibit their cytotoxicity; thus, IL‐23 inhibitors also have a synergistic effect with PD‐1 mAbs.^113^ However, the IL‐10 receptor agonist pegilodecakin could stimulate CD8+ T cell activity and induce its expansion, and its combination with PD‐1 mAbs exhibited stronger antitumor efficacy.^114^ This finding suggested that IL‐10 may have immunosuppressive and immunostimulatory effects under different conditions in RCC. The specific mechanism still needs to be further explored.

In addition, radiation therapy could positively affect cellular immunity in RCC. SABR irradiation could induce the proliferation of tumor‐responsive PD‐1+CD11a^high^CD8+ T cells in irradiated sites and drainage lymphoid tissues, and PD‐1 blockade could improve their antitumor immunity. Their combination induces the regression of unirradiated secondary tumors, known as abscopal effect, which may be caused by the migration of the abovementioned T cells with antitumor activity to the unirradiated site.^115^

Except for affecting the activation, proliferation, and function of immune cells, migration is also an important part of cellular immunity. Mavorixafor, an allosteric CXCR4 chemokine receptor inhibitor, improves immune response in patients with RCC who have no response to nivolumab. Researchers found that all patients receiving the combination therapy had elevated levels of CXCL9 chemokine expression, which could promote T‐cell activation and migration into TME.^116^, ^117^ Analysis of feces from patients with RCC treated with PD‐1/PD‐L1 mAb showed that enrichment of *A*. *muciniphila* and mucinogen was significantly associated with favorable prognosis. Experiments in vivo showed that fecal microbiota transplantation using respondent feces promoted the aggregation of CXCR3+CD4+ T cells into tumor tissues and improved the antitumor activity of PD‐1 blockade.^106^ In RCC, the low expression of adenosine and adenosine 2A receptor (A2AR) was associated with enhanced response to PD‐1 mAbs.^118^ Previous studies have shown that adenosine inhibited the activity of various antitumor immune cells by binding to A2AR on the surface of immune cells.^119^ Studies related to RCC suggested that A2AR inhibitors broadened the circulating T cell pool and promoted the recruitment of CD8+ T cells in TME.^120^

Some therapies could improve T cell activity and induce T cell recruitment simultaneously. DR‐BCAT, an RNA interference trigger, targets the *CTNNB1* gene that encodes β‐catenin. Inhibition of *CTNNB1* expression could promote an increase in the CI4 transcription level, thereby upregulating the expression of marker genes for DCs and CTLs and ultimately promoting the recruitment and cytotoxicity of CD8+ T cells.^121^ Acarbose, an alpha‐glucosidase inhibitor, could improve the efficacy of PD‐1 mAbs in the mouse model of aRCC. It could promote the recruitment of CD8+ T cells in the TME, and the activation of CD8+ T cells could be enhanced by increasing the proportion of DCs in the tumor and upregulating the expression of the costimulatory ligand CD86 on them.^122^ Vascular‐targeted photodynamic therapy, which rapidly blocks the associated blood vessels that supply tumor nutrients and leads to tumor necrosis and eradication,^123^ also improved cellular immunity in an in‐situ RENCA mouse model of lung metastatic RCC through promoting the infiltration of T cells in the metastatic site and increasing the proportion of CD8+ T cells and CD4+FOXP3‐T cells.^124^ Furthermore, cryoablation could improve T cell activity by upregulating IFN‐γ, IL‐10, and GZMB expression and promoting the infiltration of CD8+ T cells at the early stage of RCC, indicating that the combination of cryoablation and anti‐PD‐1 exhibited strong efficacy.^125^

### 4.3 Alterations of immunosuppressive cytokines and cells

The increase in immunosuppressive cytokines and the aggregation and activation of suppressive immune cells could form an “immune desert” microenvironment, which weakens the efficacy of PD‐1/PD‐L1 mAbs.^126^, ^127^, ^128^ Overexpression of IL‐1β in TME could drive tumor immune resistance.^129^ RCC‐related studies showed that anti‐IL‐1β could participate in the formation of an immunostimulatory TME through multiple mechanisms, including remodeling the medullary compartment, reducing the infiltration of polymorphonuclear myeloid‐derived suppressor cells (MDSCs) in TME, inducing macrophages to polarize to M1‐type TAMs and promoting an increase in M1‐like tumor necrosis factor, and downregulating the expression of IL‐6, CXCL8 and other immunosuppressive cytokines, in which the decrease in CXCL8 may be the potential mechanism of inhibiting the recruitment of MDSCs.^130^, ^131^ IL‐1β blockade combined with PD‐1 blockade could upregulate IFN‐γ, TNF‐α, and other inflammatory cytokines and enhance the above antitumor immune response.^131^ In the early stage of PD‐1 mAb treatment, obese mice with RCC also showed increased IL‐1β and more MDSC infiltration, and this finding is consistent with the above conclusion.^132^ Overexpression of IL‐23 in glutamine‐addicted renal carcinoma could enhance the proliferative ability and function of regulatory cells (Tregs) and thus improve the efficacy of PD‐1 blockade.^113^

The expression of signal regulatory protein α (SIRPα) expressed on macrophages, as proven to interact with CD47 on target cells to inhibit the phagocytosis of tumor cells by macrophages in breast cancer, was also abnormally increased in RCC.^133^, ^134^ Fortunately, SIRPα mAb (MY‐1) could block the above phagocytosis inhibition, induce macrophages to polarize to M1 type, and promote the accumulation of CD8+ T cells and NK cells to impair the proliferation of RENCA cells. Experiments using a colon cancer mouse suggested that MY‐1 and PD‐1 mAb have synergistic effects that could also be applied in RCC.^133^ The dysfunction of *PBRM1* in approximately 41% of patients with RCC also significantly induced the enrichment of IL‐6/JAK‐STAT and immune‐stimulating signals, which could upregulate IFN‐γ expression and may promote the formation of immune‐stimulating microenvironment, thereby improving the response to PD‐1/PD‐L1 mAbs.^135^, ^136^

Posttranslational modification is also involved in cellular immunity. Entinostat, a selective class I histone deacetylation inhibitor, significantly inhibited the immunosuppressive function of MDSCs and Treg infiltration in TME in a mouse model of renal cancer, thereby enhancing the efficacy of PD‐1 mAbs. Entinostat could significantly reduce the levels of arginase‐1 (Arg1), iNOS, and COX‐2 in MDSC to inhibit its function.^137^ Arg1 could promote L‐arginine metabolism in the circulation to induce the cell‐cycle arrest of cytotoxic T cells.^138^

## 5 COMBINATION THERAPY REGIMENS

On the basis of these potential mechanisms, several combination therapeutic regimens have been applied in fundamental experiments or clinical trials related to RCC. Here, therapies that could be combined with PD‐1/PD‐L1 mAbs and the related clinical trials in RCC were summarized. This summary could be helpful in the selection of treatment options for patients with RCC who have no response to PD‐1/PD‐L1 mAbs.

### 5.1 Classic treatment regimens

AAs or CTLA‐4 mAb plus PD‐1/PD‐L1 mAb are classic treatment regimens, some of which have been approved by the Food and Drug Administration for the treatment of aRCC. Pan‐cancer studies demonstrated that VEGF could make vascular abnormalities reduce tumor perfusion and then promote acidosis and hypoxia to form an immunosuppressive TME; it could also downregulate the expression of adhesion molecules on endothelial cells, increase interstitial fluid pressure, and upregulate PD‐1 expression on T cells to inhibit the activity and infiltration of T cells.^139^ CTLA‐4 could weaken the activity of T cells in lymph nodes and tissues by limiting the co‐stimulation of CD28 and inhibit the activity of DC cells by Treg.^140^ Not all of these mechanisms have been demonstrated in RCC, but according to the results inTable 2,^12^, ^13^, ^15^, ^16^, ^141^ these two combination therapies exhibited superior antitumor efficacy over monotherapy, although their safety remains questionable.

**TABLE 2:** Phase II or higher clinical trials using AAs or CTLA‐4 mAbs plus PD‐1/PD‐L1 mAbs

NCT,study	Phase	Experimental arm (pts,agents)	Control arm (pts,agents)	Primary endpoints	ORR (exp,%)	Drug‐related high garde AEs(exp, %)
NCT02420821 IMmotion151	III	454; atezolizumab plus bevacizumab	461; sunitinib	OS, PFS	37%, 95%CI 32–41	40.4%
NCT02853331 KEYNOTE−426	III	432; pembrolizumab plus Axitinib	429; sunitinib	OS, PFS	59.3%, 95%CI 54.5–63.9	75.8%
CheckMate 214 NCT02231749	III	550; nivolumab plus ipilimumab	546; sunitinib	OS, PFS, ORR	39.1% 95%CI 35.0–43.3	47.3%
NCT02684006 JAVELIN Renal 101	III	442; avelumab plus axitinib	444; sunitinib	OS, PFS	52.5%, 95%CI 47.7–57.2	56.7%
NCT01984242 IMmotion150	II	101; atezolizumab plus bevacizumab	101; sunitinib	PFS	32.0%	39.6%
NCT03029780 CheckMate 800	II	arm1: co‐Administration, 52 arm2: sequential Administration, 52; nivolumab Plus ipilimumab	None	AEs	NA	arm1: 36.5% arm2: 38.5%
NCT02348008 GU14‐003	II	48; pembrolizumab plus bevacizumab	None	ORR	60.9% 95%CI 45.4–74.9	NA

Phase II or higher clinical trials with AAs or CTLA‐4 mAbs plus PD‐1/PD‐L1 mAbs as interventions that had ORRs. These clinical trials only focus on patients with aRCC.

Abbreviations: AEs, adverse events; exp, experimental; NA, not available; OS, overall survival; ORR, objective response rate; PFS, progression free survival; pts, patients.

### 5.2 Other possible combination therapy options

In addition to classic combination therapies, multiple therapies could be combined with PD‐1/PD‐L1 mAbs to treat aRCC (Table 3). The mechanisms of enhancing the antitumor immunity by some therapies were mentioned above, but more potential preclinical mechanisms must be explored. Encouragingly, IL‐2 and its agonist, SABR, and inhibitors targeting multiple receptor tyrosine kinases have shown a strong potential to improve the efficacy of PD‐1/PD‐L1 mAbs, and they been used in multiple phase I clinical trials presented in Table 3. These treatments may hold the promise for patients with refractory aRCC.

**TABLE 3:** Other therapies that can be combined with PD‐1/PD‐L1 mAbs

Type	Therapy		Brief description	NCTs
Drug therapies				
Cytokines or their agonists/inhibitors	IL−2			NCT03111901, NCT03260504, NCT03991130
	ALKS 4230	Binding the intermediate affinity IL−2 receptor complex		NCT02799095
	NKTR−214	A CD122 IL−2 agonist		NCT02983045, NCT03435640, NCT03729245, NCT04540705
	Canakinumab	IL−1β mAb		NCT04028245
	Gevokizumab	IL−1β mAb		NCT03798626
	rhIL−15	Recombinant human IL−15		NCT04150562
	SO‐C101	IL−15 receptor alpha recombinant protein		NCT04234113
	N−803	IL−15 antagonist		NCT03228667
	GITR	Glucocorticoid‐induced TNF receptor‐associated proteins		NCT03126110, NCT03277352
	PegIFN−2β	Pegylated Interferon Alfa−2β		NCT02089685
	NIS793	TGF‐β inhibitor		NCT02947165
	Mogamulizuma	CCR4 mAb		NCT02946671
	X4P−001	CXCR4 inhibitor		NCT02923531
	IRX−2	A multitarget biologic agent containing physiological quantities of IL−1β, IL−2,IFNγ,TNFα, etc.		NCT03758781
Co‐inhibitory/co‐stimulatory molecules	Varlilumab	Anti‐CD27 mAb		NCT02335918, NCT02543645
	BMS−986315	Anti‐NKG2A mAb		NCT04349267
	MBG453	Tim−3 mAb		NCT02608268
	Relatlimab	LAG−3 mAb		NCT02996110
	LAG525	LAG−3 mAb		NCT02460224
	INCAGN01949	OX−40 mAb		NCT03241173
	APX005 M	CD40 agonist		NCT03502330, NCT04495257
	CDX−1140	CD40 agonist		NCT03329950
	INBRX−106	OX40 agonist		NCT04198766
Metabolism‐related molecules	Ciforadenant	Inhibitor of adenosine A2AR		NCT02655822
	NIR178	Inhibitor of adenosine A2AR		NCT03207867
	Etrumadenan	Inhibitor of adenosine A2AR		NCT03629756
	LY3475070	Inhibit CD73 to reduce adenosine production		NCT04148937
	CPI−006	Inhibit CD73 to reduce adenosine production		NCT03454451
	Oleclumab	Inhibit CD73 to reduce adenosine production		NCT04262375
	CB−839	Glutaminase inhibitor		NCT02771626
Genetic alterations	PT2385	HIF−2α inhibitor		NCT02293980
	Itacitinib	PI3Kδ inhibitor		NCT02646748, NCT02899078
	Savolitinib	c‐MET inhibitor		NCT02819596
	APL−101	c‐MET inhibitor		NCT03655613
	Sitravatinib	Target multiple RTKs, including c‐Kit, c‐Met, etc.		NCT04518046, NCT03015740, NCT03680521, NCT03680521
	Olaparib	PARP inhibitor		NCT03741426
	Niraparib	Selective PARP1 and PARP2 inhibitor		NCT04779151
	Denosumab	Receptor activator of NF‐κB ligand mAb		NCT03280667
	XmAb®18087	A bispecific antibody that recruits T cells via CD3 to kill SSTR2‐expressing tumor cells		NCT03849469
	ARRY−614	p38‐MAPK dual inhibitor		NCT04074967
Epigenetic alterations	Guadecitabine	DNA methyltransferase inhibitor		NCT03308396
Posttranslation modification	Vorinostat	HDAC inhibitor		NCT02619253
	Entinostat	HDAC inhibitor		NCT03024437, NCT03552380
	HBI−8000	HDAC inhibitor		NCT02718066
Chemotherapy	Irinotecan	DNA topoisomerase I inhibitor		NCT02423954
	Gemcitabine	DNA synthesis inhibitor		NCT03483883
	Cyclophosphamide	Cell cycle specific alkylating agent acting on the S phase		NCT04262427
Affect immunosuppressive cells	FPA008	CSF1R antibody,causing TAMs exhaustion		NCT02526017
	AZD8701	Restrict Treg function by inhibiting FOXP3		NCT04504669
	INCB001158	Arg−1 inhibitor		NCT02903914
	KY1044	kill Tregs that were highly expressed in ICOs by ADCC		NCT03829501
	Eganelisib	PI3K‐γ inhibitor targeting M2 type macrophages		NCT03961698
	GB1275	A molecule modulator of CD11B inhibiting the infiltration of TAMs		NCT04060342
Non‐drug therapies				
Radiation therapy	Radiation therapy			NCT02318771, NCT02962804, NCT02978404
	SBRT/SABR	Focusing radiotherapy of small irradiation field is realized by stereotactic and positioning technology		NCT02599779, NCT02781506, NCT02855203, NCT03693014, NCT04235777, NCT02992912, NCT03065179, NCT03115801, NCT03149159, NCT03511391
	Hypofractionated radiation therapy	Increase the dose per exposure and reduce the total number of exposures		NCT03050060
Vaccine	Ankara vaccine	Modified vaccinia virus vaccine expressing p53		NCT02432963
	RO7198457	mRNA‐based vaccines customized based on sequencing results		NCT03289962
	DSP−7888	WT1 peptide vaccine, inducing WT1‐specific CTLs and helper T cells		NCT0331133
	GEN−009 adjuvanted vaccine	A tailored vaccine customized by using autologous T cells to identify tumor neoantigens		NCT03633110
Physical ablation	Cryoablation			NCT03189186
	LITT	Laser interstitial thermal therapy		NCT04187872
Surgery	Nephrectomy or Metastasectomy			NCT02595918
	Conventional Surgery			NCT03055013
	Cytoreductive nephrectomy			NCT03977571, NCT04322955
Adoptive cell	FT516	Modified NK cells that enhances ADCC		NCT04551885
	CMN−001	Dendritic cell‐based immunotherapy		NCT04203901
	D‐CIK	a heterogeneous subset of ex vivo expanded T lymphocytes		NCT02886897, NCT03987698
Intestinal microbiota	MRx0518	Oral probiotics		NCT03637803
	EDP1503	Oral monoclonal microbial product		NCT03775850
	CBM588	Clostridium butyricum, a probiotic strain		NCT03829111
	FMT	Faecal bacteria transplantation		NCT04163289

Therapies that can be combined with PD‐1/PD‐L1 mAbs, except for AAs or CTLA‐4 mAbs. Brief descriptions of these therapies and related trials of the combination therapy are also presented in the table.

Abbreviations: A2AR, adenosine 2A receptor; ADCC, antibody‐dependent cell‐mediated cytotoxicity; Arg, arginine; CCR4, CC chemokine receptor 4; CSF1R, colony‐stimulating factor‐1 receptor; CTL, Cytotoxic T lymphocyte; CXCR4, Chemokine receptor 4; GITR, glucocorticoid‐induced TNF receptor; HDAC, histone deacetylase; HIF, hypoxia inducible factor; IFN, interferon; IL, interleukin; LAG‐3, lymphocyte activation gene 3; MAPK, mitogen‐activated protein kinases; mAb, monoclonal antibody; NF‐κB, nuclear factor kappa‐B; NKG2A, natural killer group 2A; PARP, poly(ADP‐ribose) polymerase; PI3K, phosphatidylinositol 3‐kinase; RTK, receptor tyrosine kinase; SSTR, somatostatin receptor; TAM, tumor‐associated macrophages; Treg, regulatory T cell; TNF, tumor necrosis factor.

## 6 CONCLUSIONS

This article focused on the existing mechanisms affecting the efficacy of PD‐1/PD‐L1 mAbs in aRCC. Biomarkers, such as *PBRM1* and intratumoral heterogeneity, that may predict the response to PD‐1/PD‐L1 mAbs were also discussed. According to the existing mechanisms affecting PD‐L1 expression in RCC, constitutive PD‐L1 overexpression and PD‐L1 overexpression induced by long‐term tumor neoantigen stimulation may be associated with poor response, while tumor cells stimulated by the immune microenvironment promote the secretion of inflammatory cytokines, such as IFN‐γ and IL‐1β, thus inducing adaptive PD‐L1 overexpression, which is related to enhanced response. Inflammatory cytokines may help distinguish these three situations and predict the curative effect. For patients with refractory aRCC, the clinical transformation of these fundamental mechanisms is particularly important, and the efficacy of combination therapies still needs to be further explored. Although numerous combination therapies based on PD‐1/PD‐L1 mAbs have been applied in clinical trials, their clinical prevalence still has a long way to go.

## 7 CONFLICT OF INTERESTS

The authors declare that they have no competing interests.

## Data Availability

All data generated or analyzed during this study are included in references or can be found in clinicaltrials.gov.
